# Myosteatosis rather than sarcopenia associates with non‐alcoholic steatohepatitis in non‐alcoholic fatty liver disease preclinical models

**DOI:** 10.1002/jcsm.12646

**Published:** 2020-11-26

**Authors:** Maxime Nachit, Maxime De Rudder, Jean‐Paul Thissen, Olivier Schakman, Caroline Bouzin, Yves Horsmans, Greetje Vande Velde, Isabelle Anne Leclercq

**Affiliations:** ^1^ Laboratory of Hepato‐Gastroenterology, Institute of Experimental and Clinical Research UCLouvain Brussels Belgium; ^2^ Department of Imaging and Pathology KU Leuven Leuven Belgium; ^3^ Pole of Endocrinology, Diabetes and Nutrition, Institute of Experimental and Clinical Research UCLouvain Brussels Belgium; ^4^ Institute of Neuroscience UCLouvain Brussels Belgium; ^5^ IREC Imaging Platform UCLouvain Brussels Belgium; ^6^ Service d'Hépato‐Gastro‐Entérologie Cliniques Universitaires Saint‐Luc Brussels Belgium; ^7^ Molecular Small Animal Imaging Center (MoSAIC) KU Leuven Leuven Belgium

**Keywords:** NAFLD, Steatohepatitis, Muscle density, Myosteatosis, Sarcopenia, Muscle, Muscle fat, Micro‐CT, Obesity

## Abstract

**Background:**

Non‐alcoholic fatty liver (NAFL) disease (NAFLD) is the most common chronic liver disease in the world. While most subjects have ‘inert’ NAFL, a subset will progress to non‐alcoholic steatohepatitis (NASH) and its life‐threatening complications. A substantial body of literature supports that a low muscle mass, low strength, and/or muscle fatty infiltration (myosteatosis) are associated with NAFLD severity. Here, we evaluated the muscle compartment in NASH preclinical models to decipher the kinetics of muscle alterations in relation with liver disease progression.

**Methods:**

We developed and validated a micro‐computed tomography‐based methodology to prospectively study skeletal muscle mass and density in muscle and liver (i.e. reflecting fatty infiltration) in a high‐throughput and non‐invasive manner in three preclinical NAFLD/NASH rodent models: fat aussie (FOZ) mice fed a high‐fat diet (FOZ HF), wild‐type (WT) mice fed a high‐fat high‐fructose diet (WT HFF), and WT mice fed a high‐fat diet (WT HF). We compared them with WT mice fed a normal diet (WT ND) used as controls.

**Results:**

‐FOZ HF with fibrosing NASH had sarcopenia characterized by a reduced muscle strength when compared with WT HF and WT HFF with early NASH and WT ND controls (165.2 ± 5.2 g vs. 237.4 ± 11.7 g, 256 ± 5.7 g, and 242.9 ± 9.3 g, respectively, *P* 60; 0.001). Muscle mass or strength was not lower in FOZ HF, WT HF, and WT HFF with early NASH than in controls. Myosteatosis was present in FOZ HF with fibrosing NASH, but also in FOZ HF, WT HF, and WT HFF with early NASH (muscle density = 0.50 ± 0.02, 0.62 ± 0.02, 0.70 ± 0.05, and 0.75 ± 0.03, respectively, with *P* 60; 0.001 when compared with respective controls). Myosteatosis degree was strongly correlated with NAFLD activity score (*r* = −0.87, *n* = 67, *P* 60; 0.001). In multivariate analysis, the association between myosteatosis and NASH was independent from homeostatic model assessment of insulin resistance and visceral fat area (*P* 60; 0.05). Myosteatosis degree powerfully discriminated NASH from benign NAFL and normal liver (area under the receiver operating characteristic = 0.96, *n* = 67, *P* 60; 0.001).

**Conclusions:**

Taken together, our data support that there is no sarcopenia in obese mice with early NASH. In contrast, the severity of myosteatosis reflects on hepatocellular damage and inflammation during early NASH development. This observation prompts us to exploit myosteatosis as a novel non‐invasive marker of NASH.

## Introduction

Non‐alcoholic fatty liver (NAFL) disease (NAFLD) is the most common chronic liver disease in the world.^1^, ^2^, ^3^ NAFLD encompasses a spectrum of diseases ranging from NAFL, affecting 25% of the world adult population,^1^, ^3^ to non‐alcoholic steatohepatitis (NASH) that will sooner or later lead to fibrosis and eventually to end‐stage liver disease and hepatocellular cancer.^1^ Today, except if severe fibrosis is present, tools are lacking to identify among NAFLD patients those with NASH and thus at risk of progressive liver disease.^1^, ^3^ Examination of a liver biopsy is the only mean to diagnose the transition.^4^

Almost all chronic diseases are associated with alterations of the muscle compartment. A low muscle mass, low strength, and/or severe muscle fatty infiltration (often designated as myosteatosis) are clearly recognized as powerful indicators of the disease prognosis.^5^ Liver diseases are no exception. A broad literature has already linked sarcopenia^6^, ^7^ (used abusively as a synonym for mere low muscle mass in most liver studies) and myosteatosis to prognosis in cirrhosis.^8^, ^9^, ^10^, ^11^ Hence, it is now suggested to take into account muscle parameters in the evaluation of patients for liver transplantation.^9^, ^12^, ^13^ Highly cited studies have expanded on this literature with strong data supporting the presence of muscle alterations in NAFLD patients with liver fibrosis.^11^, ^14^, ^15^, ^16^, ^17^, ^18^, ^19^, ^20^, ^21^, ^22^ They reported that a low muscle mass, low strength and/or myosteatosis are associated with fibrosis severity in NAFLD.^11^, ^14^, ^15^, ^16^, ^17^, ^18^, ^19^, ^20^, ^21^, ^22^, ^23^ Nonetheless, whether muscle alterations are mere consequences of severe liver dysfunction or whether they precede or accompany liver disease progression is unknown. These questions generate a large interest in the field of NAFLD.^11^, ^19^, ^24^ We need longitudinal data to address such hypotheses. However, because of the long time‐course of NAFLD progression in humans and the lack of muscle data at asymptomatic early disease stages, it is unrealistic to obtain such data in humans.

To unravel the sequence of events, we therefore characterized muscles and liver in three validated preclinical models of NAFLD/NASH. We used high‐fat (HF) diet‐fed fat aussie (FOZ) (FOZ HF), HF high‐fructose diet‐fed wild‐type (WT) mice (WT HFF), and HF diet‐fed WT mice (WT HF). We purposedly neglected models with amino acid‐deficient diets or genetic models that have innate disrupted leptin signalling to exclude potential bias for muscle compartment alterations.^19^, ^25^ FOZ HF, WT HFF, and WT HF mice exhibit obesity, insulin resistance, liver steatosis, and recapitulate key histological NASH features after 4, 16, and 34 weeks of diet, respectively. With this study, we discovered that sarcopenia is not present in early NASH. In contrast, severe muscle fatty infiltration (i.e. myosteatosis) was a consistent, specific, and early marker of NASH in three preclinical models. Thus, our data foster the exploitation of myosteatosis as a novel non‐invasive indicator of NASH in NAFLD.

## Materials and methods

### Animal and diets

The FOZ mouse strain on a non‐obese diabetic mice B10 background^26^ was bred and maintained at a constant temperature of 22°C and exposed at all times to a 12 h light/12 h dark cycle. Heterozygous mice (−/+) were used for breeding and homozygous FOZ (−/−) (*n* = 48) and WT (+/+) (*n* = 89) male littermates for the dietary experiments. Soon after weaning (at 5 weeks of age noted as Time 0 in our timeline) mice were turned on a HF diet (60% of calories from fat, 0.03% cholesterol—Research Diets D12492), HF diet supplemented with 30% fructose in drinking water (HFF) or kept on a rodent chow [normal diet (ND), 10% of calories from fat; Carfil Quality, Oud‐Turnhout, Belgium] (Supporting information, *Figure* S1A). At Time 0 and once a month, we recorded body weight, glycaemia and insulinemia; we measured food intake; and we performed a whole‐body computed tomography (CT) scan to evaluate the muscle and liver compartment non‐invasively. Groups of *n* = 5–9 mice were sacrificed at relevant time points. At the time of sacrifice, mice were anaesthetised (ketamine/xylazine), and blood was withdrawn by cardiac puncture. Liver and muscles (tibialis anterior, extensor *digitorum longus*, soleus, gastrocnemius, quadriceps, and erector *spinae*/*quadratus lumborum*) were quickly harvested and weighted; and samples were snap frozen in liquid nitrogen and stored at −80°C until analyses, fixed in 4% formalin and embedded in paraffin (liver and muscles) or directly embedded in optimal cutting temperature compound, and frozen for histological analyses. FOZ ND data are shown in*Figure* S2 for information but will not be presented hereafter for simplification as they exhibited the same liver and muscle phenotype as WT HF. All experiments have been performed in accordance with the Animal Research: Reporting of In Vivo Experiments guidelines. Anthropometric data and detail histological analysis of the animals of this cohort have been published in De Rudder *et al*.^27^ The animals were handled according to the guidelines for humane care of laboratory animals established by the *Université catholique de Louvain* in accordance with European regulations, and the study protocol was approved by the university ethics committee (2016/UCL/MD/003).

### Metabolic parameters and biochemical analyses

Glucose and insulin levels were monthly monitored on tail blood using a glucometer (Accu‐Chek) and a commercial ELISA test (Mercodia AB, Sweden), respectively. Homeostatic model assessment of insulin resistance (HOMA‐IR) was calculated as [glycaemia (mmol/L) × insulinemia (mU/L)]/22.5.^28^ Food consumption was measured over a 7 day period once a month and divided by the number of animals in the cage (*n* = 5–6). We reported energy intake in kilocalorie per mouse per day. Lipids were extracted from 50 mg of liver or 100 mg of muscle (erector *spinae*/*quadratus lumborum*) using methanol and chloroform, and total lipids were quantitated using the vanillin–phosphoric acid reaction.^29^ Results were normalized on weighted muscle or liver.

### Micro‐computed tomography

Once a month, we performed micro‐CT on mice anaesthetized with isoflurane to monitor changes in body composition, skeletal muscle mass, skeletal muscle, and liver fatty infiltration. Scanning was performed with a Skyscan 1278 (Bruker micro‐CT, Kontich, Belgium) at 50 μm voxel resolution using a source voltage of 65 kV and a current of 770 μA. Aluminium filter was set on 1 mm to optimize contrast while minimizing dose. Rotation step for the X‐ray source was set on 0.7°. Average scanning time per animal was 2.5 min, allowing for very high throughput. Raw images were then reconstructed with NRecon to 3D cross‐sectional image data sets using the following parameters: beam hardening to 10%, smoothing to 2%, minimum for CS to Image Conversion to 0%, maximum to 0.02%. Analyses of reconstructed images were performed using SkyScan software (CTan), and segmentation of different tissue compartments was based on specific tissue density in Hounsfield units (HU). Three types of analyses were performed on micro‐CT reconstructed data sets. Whole body analysis: whole body fat volume, whole body lean volume (muscles and organs), and whole‐body bone volume, all reported in cubic centimetre. μCT‐estimated body weight was computed by adding the volumetric density of fat free mass (1.05 g/cm^3^), fat mass (0.95 g/cm^3^), and bone mass (1.92 g/cm^3^) multiplied by their measured volume.^30^ Single slice‐based analysis: The surface (mm^2^) and density (in HU) of erector *spinae*/*quadratus lumborum* and psoas muscle, here designed as ‘dorsal muscle’, were semi‐automatically measured. First, two regions of interest were manually drawn on the dorsal muscle area at L4 and L5. Then, a grey value threshold (30 to 97) was applied to exclude non‐lean tissue (i.e. mostly bone). Finally, muscle area and density (transformed in HU) measures at L4 and L5 were saved and respectively averaged. We computed the relative dorsal muscle area by normalizing dorsal muscle area (mm^2^) by body weight (g). Visceral fat was semi‐automatically measured at L4 using manual region of interest delineation and grey value threshold (30 to 57). Virtual liver biopsy was performed by placing a 3D cylindrical region of interest (±1.3 cm^3^) in the liver avoiding large vessels, and the mean density (HU) of the entire volume was automatically computed. In each animal, dorsal muscle and liver density were normalized to spleen density (an internal invariant control). The muscle‐to‐spleen ratio and the liver‐to‐spleen ratio are referred to as the muscle and the liver density, respectively.

### Muscle strength

A grip strength test measured the combined strength of the forelimb and hindlimb using a grid connected to a sensor (Panlab‐Bioseb, Vitrolles, France). The mice were gently laid on the top of a grid inclined at 45° and were pulled back steadily until the grip was released down the complete length of the grid. Force was tested three to five times sequentially and retested again 20 min later. Results are presented as the mean of the two highest values of absolute force recorded for each test.

### Histology, immunohistochemistry, and immunofluorescence

Formalin‐fixed paraffin‐embedded sections stained with haematoxylin and eosin or Sirius red were used for histological evaluation of the liver and NAFLD activity score (NAS), blindly assessed as per Kleiner *et al*.,^31^ or for fibrosis assessment, respectively. Fibrosis area was automatically assessed using a dedicated software (Biocellvia, France) and expressed as the ratio of the area of stained collagen fibres vs. the area of the liver section in percentage as reported for this cohort in De Rudder *et al*.^27^

Non‐alcoholic steatohepatitis diagnosis was defined according to the SAF algorithm; thus, samples with NAS ≥ 3 with at least 1 point in each sub‐score (i.e. steatosis, inflammation, and ballooning) were considered as NASH while those with at least 1 point in steatosis were considered as NAFL.^32^

Wheat‐germ agglutinin (RL‐1022, Vector) was immunodetected on 4 μm thick paraffin sections of quadriceps muscle to measure fibre size. Slides were scanned using Pannoramic 250 Flash III scanner (3DHISTECH), and the fibre00027;s size was measured on the entire sections using an in‐house macro on the image analysis tool Author Version 2017.2 (Visiopharm, Denmark).

### Statistics

All data are presented as mean ± SEM. Statistical analyses were performed using a two‐tailed Student00027;s *t* test, a one‐way analysis of variance (ANOVA), or a two‐way ANOVA (mixed model) followed by Bonferroni00027;s *post‐hoc* using GraphPad Prism 8 software. Multivariate analyses were performed on SPSS (v24) using binary logistic regression. Differences were considered significant at values of *P* 60; 0.05.

## Results

### Sarcopenia associates with fibrosing non‐alcoholic steatohepatitis, but not with early non‐alcoholic steatohepatitis

**FIGURE 1:**
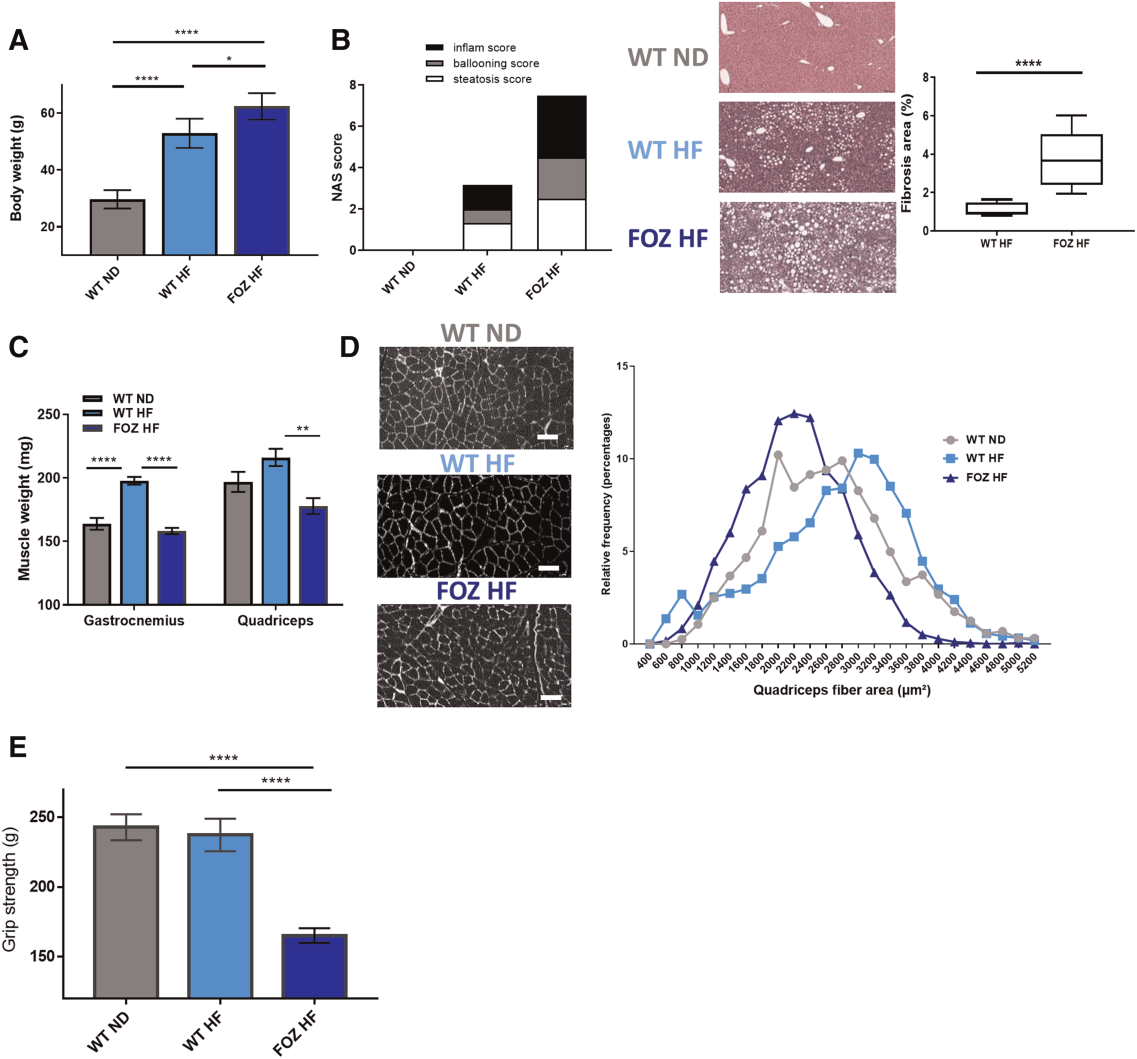
Fibrosing non‐alcoholic steatohepatitis is associated with severe muscle alterations in high‐fat (HF) diet‐fed fat aussie (FOZ HF) mice. *(A)* Body weight of wild‐type (WT) normal diet (WT ND), WT HF, and FOZ HF mice after 34 weeks of diet (*n* = 6 WT ND, *n* = 5 WT HF, *n* = 5 FOZ HF, one‐way ANOVA). *(B)* Left, histological non‐alcoholic fatty liver disease activity score (NAS) performed on haematoxylin and eosin (H38;E)‐stained liver sections. Center, representative histological pictures, scale bar = 100 μm. Right, fibrosis area percentage calculated on entire liver sections (*n* = 3–4 per group). Line, median value; box, 25–75% percentile; whiskers, min and max. *(C)* Gastrocnemius and quadriceps muscle weight in WT ND, WT HF, and FOZ HF (*n* = 6 WT ND, *n* = 5 WT HF, *n* = 5 FOZ HF, one‐way ANOVA). *(D)* Left, paraffin cross‐section of quadriceps with myofibers stained with wheat‐germ agglutinin (WGA). Scale bar = 50 μm. Right, histogram of relative frequency distribution (%) of myofibers size (WT ND *n* = 4, mean = 2632.8 ± 20.3 μm^2^ and median = 2579 μm^2^; WT HF *n* = 4, mean = 2749 ± 19.5 μm^2^ and median = 2858.5 μm^2^; FOZ HF *n* = 3, mean = 2221.8 ± 14.9 μm^2^ and median = 2219.3 μm^2^). WT ND, WT HF, and FOZ HF mean fibre sizes are all significantly different from each other with *P* 60; 0.0001 (one‐way ANOVA). *(E)* Absolute grip strength (*n* = 4–9 animals per group, one‐way ANOVA). All data are mean ± SEM. *P 60; 0.05, **P 60; 0.01, ***P 60; 0.001, ****P 60; 0.0001.

After 34 weeks of HF diet, WT and FOZ had severe obesity (52.9 ± 2.3 g and 62.4 ± 2.1 g, respectively) (*Figure* 1A) associated with early NASH in WT HF and with fibrosing NASH in FOZ HF mice (*Figures* 1B and S3A,B). Chow‐fed WT (WT ND) used as controls had a normal weight and liver. FOZ HF mice with fibrosing NASH had lower muscle mass than WT HF with early NASH (*Figure* 1C). Accordingly, the size of the muscle fibres was smaller in quadriceps of FOZ HF (median size 2219.3 μm^2^) compared with WT HF (median size 2858 μm^2^) (*Figure* 1D). Muscle mass was similar in FOZ HF with NASH and in controls (*1C,D*. Muscle strength was 30% lower in FOZ HF (165.2 ± 5.2 g) than in WT HF (237.4 ± 11.7 g) (*Figure* 1E). Obese WT mice on a HF diet had increased muscle mass (*Figure* 1C) and muscle fibre size (*Figure* 1D), but similar grip strength than chow‐fed controls (*Figure* 1E). Hence, muscle strength was preserved at the expense of increased muscle mass in WT HF. Thus, low muscle mass and strength are not present in early NASH but may rather associate with severe fibrotic liver disease.

### Micro‐computed tomography is an effective tool to evaluate body composition, liver steatosis, and muscle alterations non‐invasively

**FIGURE 2:**
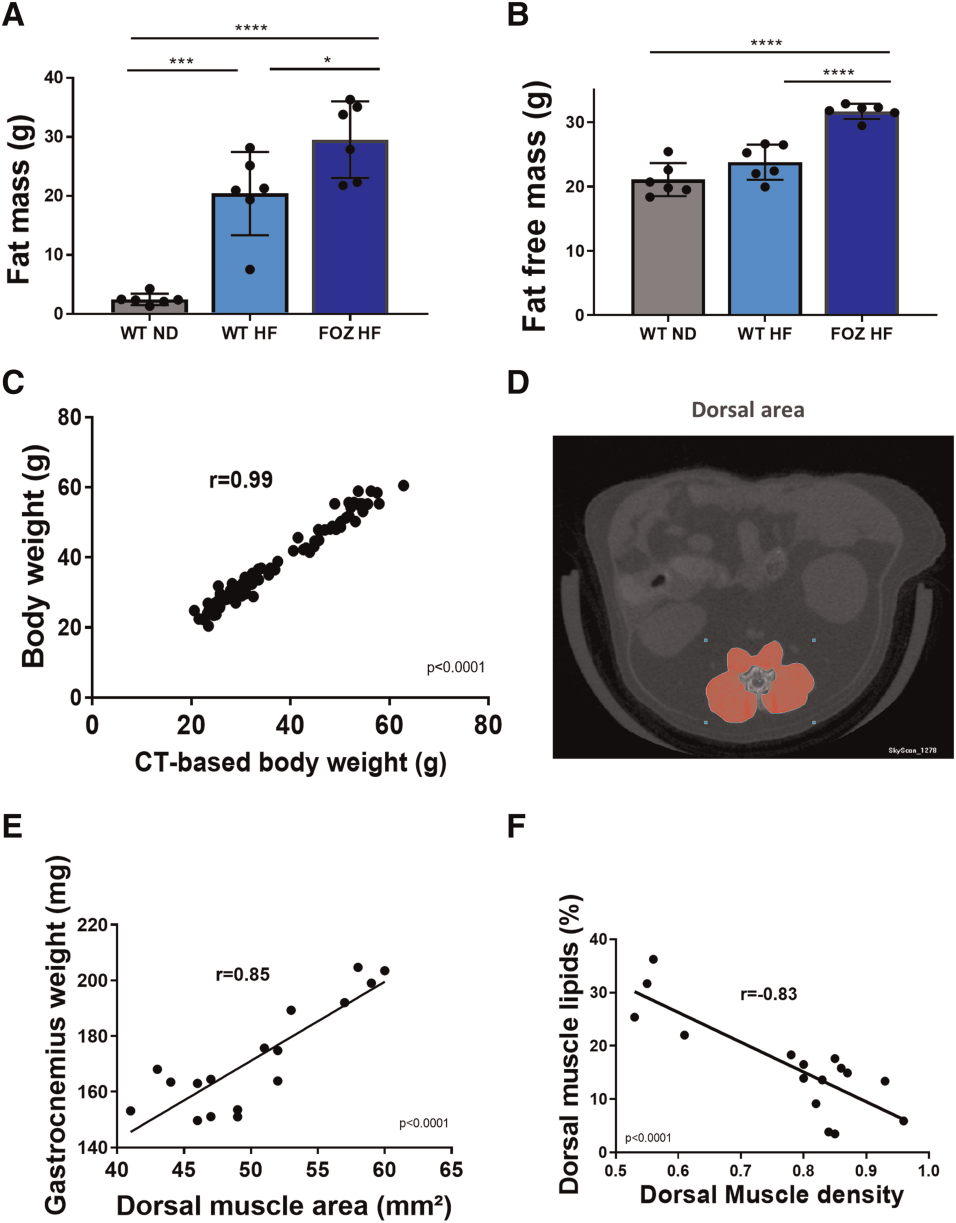
Micro‐computed tomography (CT) allows for non‐invasive ‘all‐in‐one’ evaluation of body composition, muscle mass, myosteatosis, and liver steatosis. *(A)* Fat mass and *(B)* fat‐free mass (i.e. lean body mass including muscles and organs, bones excluded) of wild‐type normal diet (WT ND), WT high‐fat‐(HF) fed (WT HF), and HF diet‐fed fat aussie (FOZ HF) mice at W34 (*n* = 6 per group, one‐way ANOVA). *(C)* Correlation between body weight and micro‐CT‐based body weight computed by adding fat‐free mass, fat mass, and bone mass measured from reconstructed CT acquisitions (Pearson00027;s coefficient *r* = 0.99, *n* = 109, *P* 60; 0.0001). *(D)* Illustration of dorsal muscle area region of interest (ROI) at L4 on micro‐CT acquisitions *(E)* correlation between dorsal muscle area (L4 and L5 averaged) and gastrocnemius muscle weight (Pearson00027;s coefficient *r* = 0.85, *n* = 18, *P* 60; 0.0001). *(F)* Correlation between dorsal muscle density (L4 and L5 averaged) and lipid content in dorsal muscle (biochemical measure) (Pearson00027;s coefficient *r* = −0.83, *n* = 16, *P* 60; 0.0001). All data are mean ± SEM, except indicated otherwise. *P 60; 0.05, **P 60; 0.01, ***P 60; 0.001, ****P 60; 0.0001.

To evaluate the kinetics of muscle alterations in relation to liver disease progression in NAFLD mouse models, we searched for a non‐invasive methodology to enable longitudinal follow‐up of liver and muscles changes. We chose micro‐CT as this tool has previously been validated for body composition measurements (i.e. fat free, fat, and bone mass) in rodent models.^33^ Of note, we previously reported that micro‐CT derived liver densities accurately reflect liver lipid content and steatosis.^27^ We first used micro‐CT to measure whole body fat mass and fat‐free mass (*Figure*s 2A,B and S4) and found an excellent agreement between micro‐CT‐estimated and measured body weight (*r* = 0.99, *P* 60; 0.0001) (*Figure* 2C). Fat mass and fat‐free mass (*Figure* 2A,B) were higher in FOZ HF than in WT HF at W34. Drawing inspiration from the clinical gold standard,^6^, ^7^, ^34^ we then measured the surface and the density of dorsal muscles (erector *spinae*, *quadratum lumborum*, and psoas) at the fourth and fifth lumbar levels (L4 and L5) (*Figure* S5) as surrogates for muscle mass and fatty infiltration, respectively. The technique has never been applied to micro‐CT scan and small animals up to now. Therefore, to validate it, we correlated dorsal muscle area at L4 and L5 (averaged) (*Figure* 2D) with gastrocnemius weight. We found a high positive correlation (*r* = 0.85) (*Figure* 2E). We then correlated dorsal muscle density with dorsal muscle lipid content quantitated by a biochemical method and found a high negative correlation (*r* = −0.83) (*Figure* 2F). Hence, dorsal muscle area and density adequately reflect muscle mass and specific dorsal muscles fatty infiltration (i.e. myosteatosis), respectively.

**FIGURE 3:**
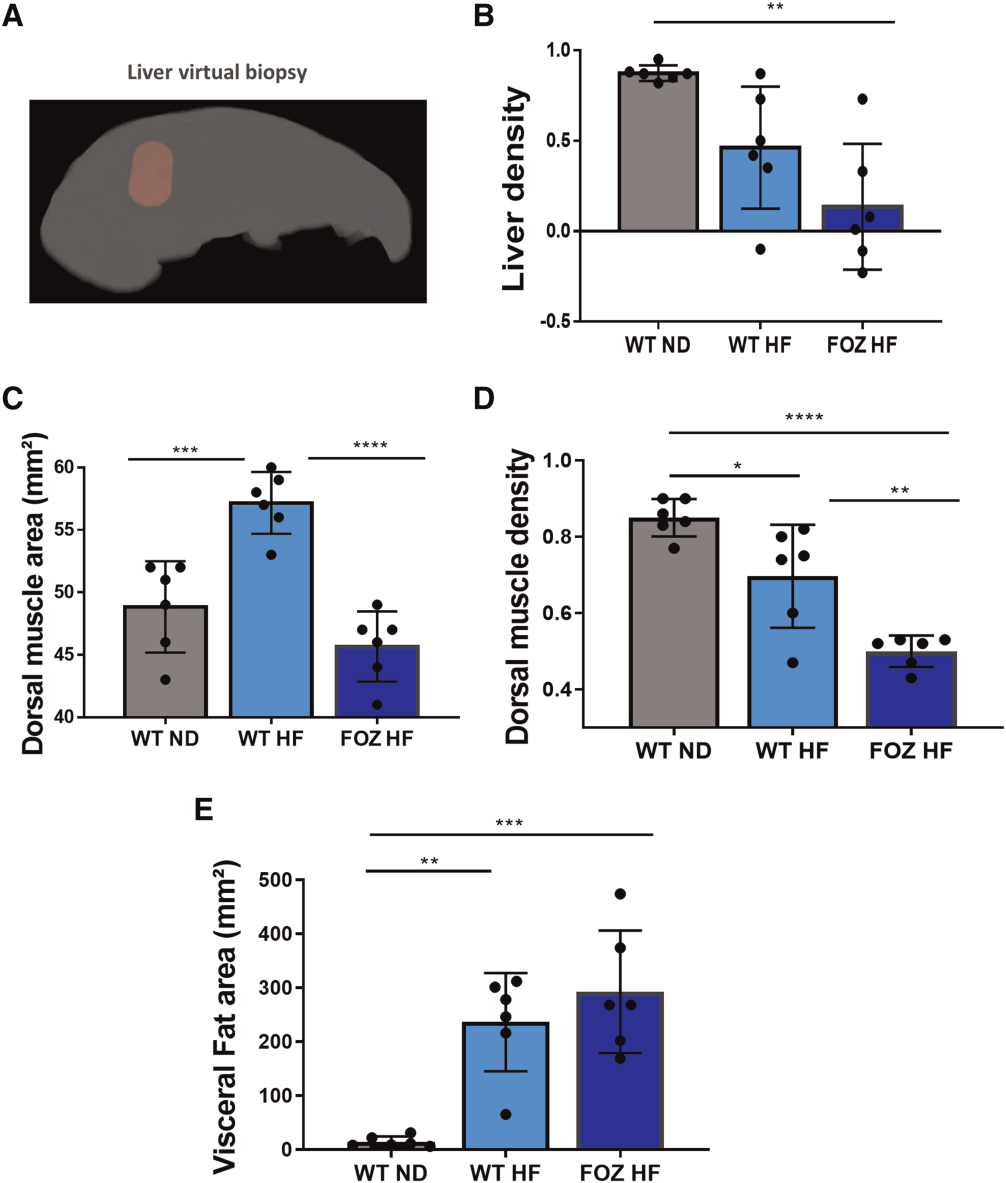
Muscle density but not muscle mass decreases according to liver disease severity. *(A)* Illustration of 3D liver biopsy as a cylindrical region of interest within the liver parenchyma avoiding large vessels for measuring liver density. *(B)* Liver density values in wild‐type normal diet fed mice(WT ND), WT high‐fat (HF)‐fed mice (WT HF), and HF diet‐fed fat aussie mice (FOZ HF) (*n* = 6 per group) after 34 weeks of diet (one‐way ANOVA). *(C)* Dorsal muscle area of WT ND, WT HF, and FOZ HF after 34 weeks of diet (one‐way ANOVA). *(D)* Dorsal muscle density of WT ND, WT HF, and FOZ HF after 34 weeks of diet (one‐way ANOVA). *(E)* Visceral fat area (at L4) of WT ND, WT HF, and FOZ HF after 34 weeks of diet (one‐way ANOVA). All data are mean ± SEM, except indicated otherwise. *P 60; 0.05, **P 60; 0.01, ***P 60; 0.001, ****P 60; 0.0001.

We then performed micro‐CT scan in WT ND, WT HF, and FOZ HF at W34 to measured liver densities using 3D liver biopsy (*Figure* 3A, see Materials and Methods). We found equally low liver densities in WT HF and FOZ HF (*Figure* 3B). Dorsal muscle area was higher in WT HF than in controls and substantially lower in FOZ HF than in WT HF (*Figure* 3C), a result that parallels changes in gastrocnemius and quadriceps muscle mass (*Figure* 1C). Interestingly, dorsal muscle density was low in HF‐fed animals and in particular, significantly lower in FOZ HF than in WT HF (*Figure* 3D). Visceral fat area measured at L4 was not different between FOZ HF and WT HF (*Figure* 3E).

Thus, micro‐CT is a suitable tool to assess liver fat and the muscle compartment non‐invasively in mice. Our data further support that early NASH is not associated with low muscle mass. In contrast and remarkably, myosteatosis (reflected by a low dorsal muscle density) is found in both early and fibrosing NASH, and its degree seems related to liver disease severity (*Figures* 1B and 3D).

### Myosteatosis is the earliest muscle alteration in mice with non‐alcoholic steatohepatitis

**FIGURE 4:**
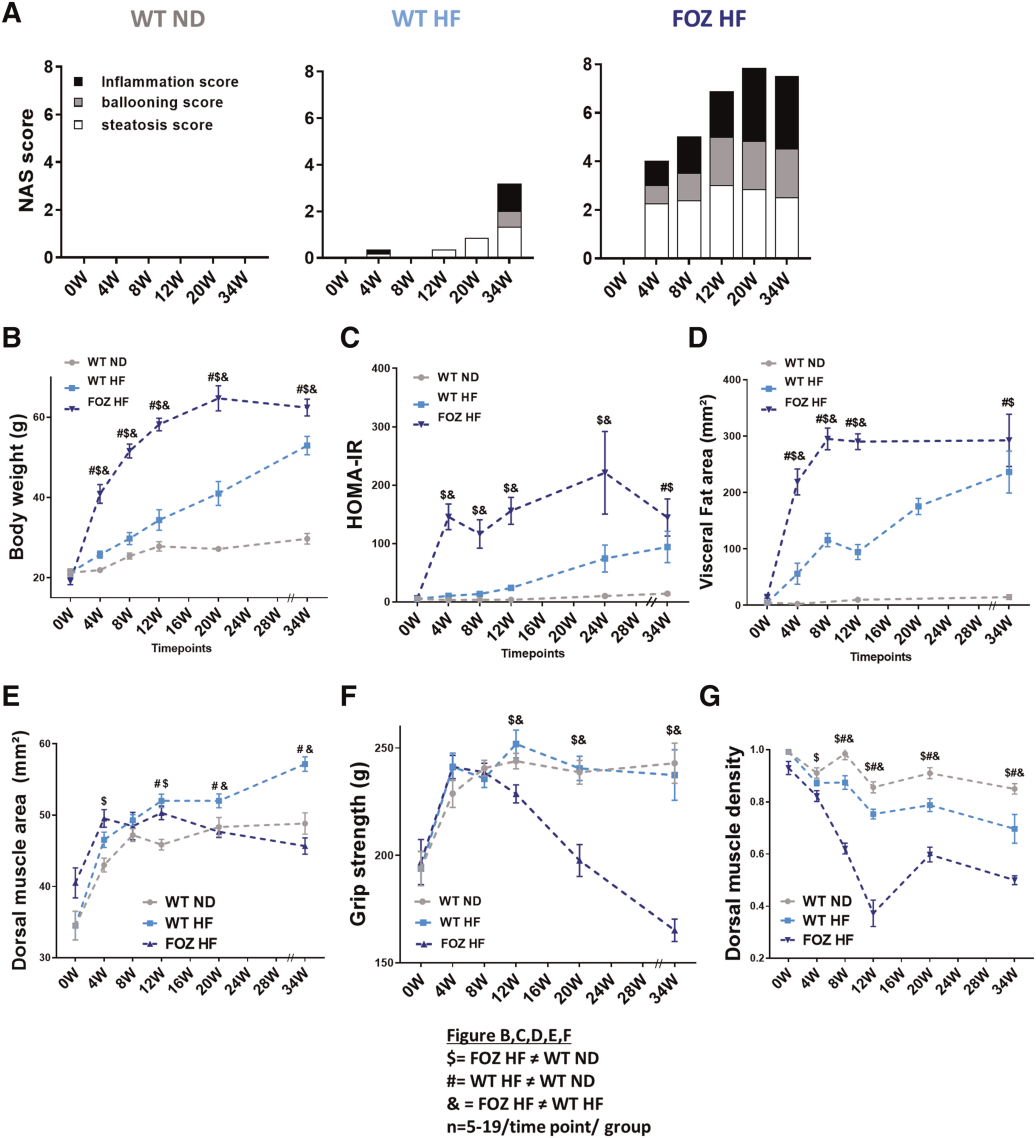
Myosteatosis is the earliest muscle alteration in mice with non‐alcoholic steatohepatitis. *(A)* Non‐alcoholic fatty liver disease activity score (NAS) of wild‐type normal diet (WT ND), WT high‐fat‐(HF) fed (WT HF), and HF diet‐fed fat aussie (FOZ HF) mice according to time point studied (*n* = 5–9 per group per time point). *(B)* Body weight, *(C)* homeostatic model assessment of insulin resistance (HOMA‐IR), *(D)* visceral fat area (L4), *(E)* dorsal muscle area (L4 and L5 averaged), *(F)* grip strength, and *(G)* dorsal muscle density (L4 and L5 averaged) measured *in vivo* by micro‐CT in WT ND, WT HF, and FOZ HF during feeding experiment (*n* = 5–19 per group per time point, two‐way ANOVA: $ = FOZ HF significantly different from WT ND; # = WT HF significantly different from WT ND; 38; = FOZ HF significantly different from WT HF). W, weeks.

To decipher the time‐course of muscle alterations in relation to liver disease progression, we conducted a longitudinal study in FOZ and WT mice fed a HF or a ND over a 34 week study period. At selected time points, we performed a micro‐CT to investigate the dorsal muscle and the liver non‐invasively and sacrificed a subset of mice to harvest tissues. As already reported in this cohort,^27^ WT ND had normal liver histology at all times; WT HF developed modest steatosis during most of the study period, but early NASH at W34; all FOZ HF had NASH from W8 on, with minimal fibrosis at W20 and patent pericellular fibrosis at W34 (*Figures* 4A and S3A,B). Weight gain was slower in WT HF than in FOZ HF, but animals in both groups reached severe obesity at W34 (*Figure* 4B). FOZ HF consumed slightly more calories than WT HF and WT ND (*Figure* S1B). HOMA‐IR was significantly higher in FOZ HF and WT HF than in WT ND from W4 on (*Figure* 4C). Visceral fat area increased in WT HF and in FOZ HF from W4 on, albeit at a slower rate in WT HF than in FOZ HF. Nonetheless, animals in both group reached similar visceral fat area at W34 (*Figure*s 3E and 4D).

Interestingly, in WT mice, high fat feeding caused an increase in muscle mass in parallel with body weight gain (*Figure* 4E). This was not seen in FOZ HF, although being overtly obese. Hence, relative muscle mass was lower in FOZ HF than in WT HF and WT ND at all time points (*Figure* S6A). Also, muscle mass of FOZ HF was similar if not higher to that of WT ND control mice (*Figures* 4E and S6B) and did not vary during the study period in spite of the progression of liver disease up to severe NASH, in opposition with the human literature.^14^, ^15^, ^16^, ^18^ Muscle strength similarly increased in all groups up to W8 (*Figure* 4F), compatible with animals00027; growth, but steeply decreased only in FOZ HF thereafter. Hence, early NASH (as in FOZ HF at W4 and W8 or WT HF at W34) did not associate with loss of muscle mass or strength. In contrast, muscle density was significantly lower in FOZ HF than in WT ND and WT HF as from W4 and W8, respectively (*Figure* 4G). Thus, myosteatosis is the earliest muscle alteration in FOZ HF mice that have NASH.

### The association between non‐alcoholic steatohepatitis and myosteatosis is not model specific and is independent from visceral fat area and insulin resistance

**FIGURE 5:**
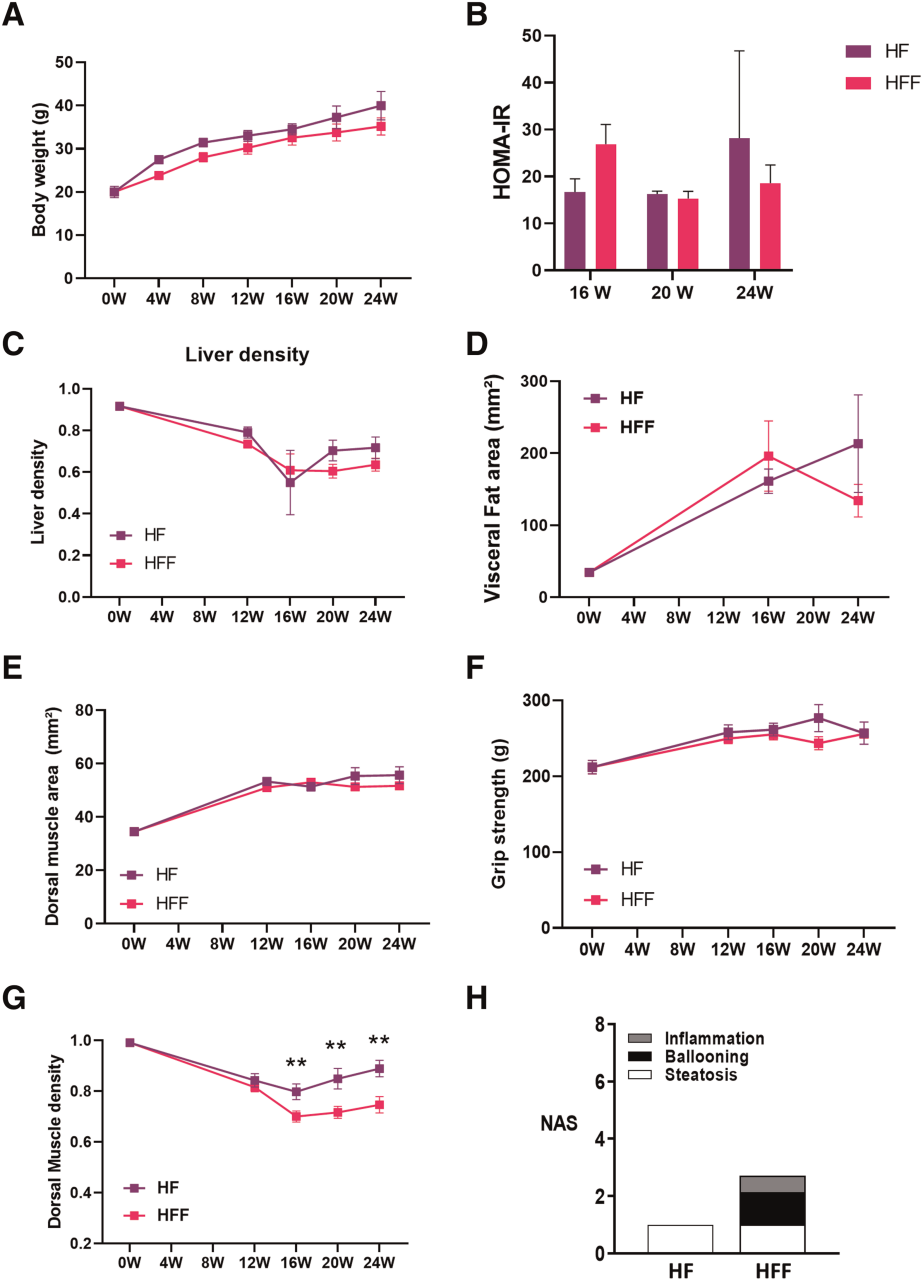
The association between non‐alcoholic steatohepatitis and myosteatosis is not model specific. *(A)* Body weight, *(B)* homeostatic model assessment of insulin resistance (HOMA‐IR), *(C)* liver steatosis, and *(D)* visceral fat area (L4), *(E)* dorsal muscle area (L4 and L5 averaged) measured *in vivo* by micro‐CT, *(F)* grip strength, and *(G)* dorsal muscle density in high‐fat (HF) diet‐fed wild‐type (WT) mice (WT HF) and HF high‐fructose diet‐fed WT mice (WT HFF) during feeding experiment (*n* = 4–11 per group per time point, two‐way ANOVA, *P 60; 0.05). *(H)* Non‐alcoholic fatty liver disease activity score (NAS) of WT HF and WT HFF at sacrifice. All data are mean ± SEM, *P 60; 0.05, **P 60; 0.01, ***P 60; 0.001, ****P 60; 0.0001. W, weeks.

NASH is present in WT HF at W34 and FOZ HF from W8 on. These mice are severely obese and insulin resistant, two conditions that could cause myosteatosis independently from liver disease severity. To verify whether our findings are not the mere reflection of a severe dysmetabolic status, we evaluated the muscle compartment in WT mice fed a high‐fat diet supplemented with fructose in drinking water (WT HFF), a regimen shown to cause NASH with 24 weeks,^25^ and compared them with WT HF controls (NAFL). The mice in the two groups were perfectly matched for calorie intake (*Figure* S7A), body weight (*Figure* 5A), HOMA‐IR (*Figure* 5B), fasting glycaemia (*Figure* S7B), and liver steatosis (*Figures* 5C and S7C) and visceral fat area (*Figure* 5D). Muscle mass (*Figure* 5E) and muscle strength (*Figure* 5F) were similar in the two groups. Remarkably, only muscle density differed between WT HFF and WT HF with lower muscle density in WT HFF than in WT HF (*Figure* 5G). We analysed the liver histology at sacrifice and found key NASH features (i.e. inflammation and ballooning), but not fibrosis, in WT HFF (*Figures* 5H and S7D). WT HF exhibited simple steatosis. Hence, the lower muscle density in WT HFF was the only parameter able to discriminate early NASH from NAFL in this model.

We then evaluated whether the association between myosteatosis and NASH was independent from insulin resistance and visceral fat area, two potential confounding factors (*Table* S1). We used multivariate analysis in the pooled animal cohort from the two experiments (WT ND, WT HF, FOZ HF, and WT HFF, total *n* = 84). In univariate analysis, muscle density was a significant predictor of NASH whether all animal were considered (*n* = 84) or only those with NAFLD (*n* = 45) (Table S1). We then adjusted the analysis for HOMA‐IR and visceral fat area, available in *n* = 53 animals of the pooled cohort (*n* = 31 in animals with NAFLD). Remarkably, muscle density remained the only significant predictor of NASH (*Table* S1). These data strongly support our key messages, that are, that there is no sarcopenia in early NASH and that the association between myosteatosis and NASH is related to liver disease severity and is not explained by the metabolic milieu.

### Myosteatosis strongly discriminates non‐alcoholic steatohepatitis from fatty liver in preclinical models of non‐alcoholic fatty liver disease

**FIGURE 6:**
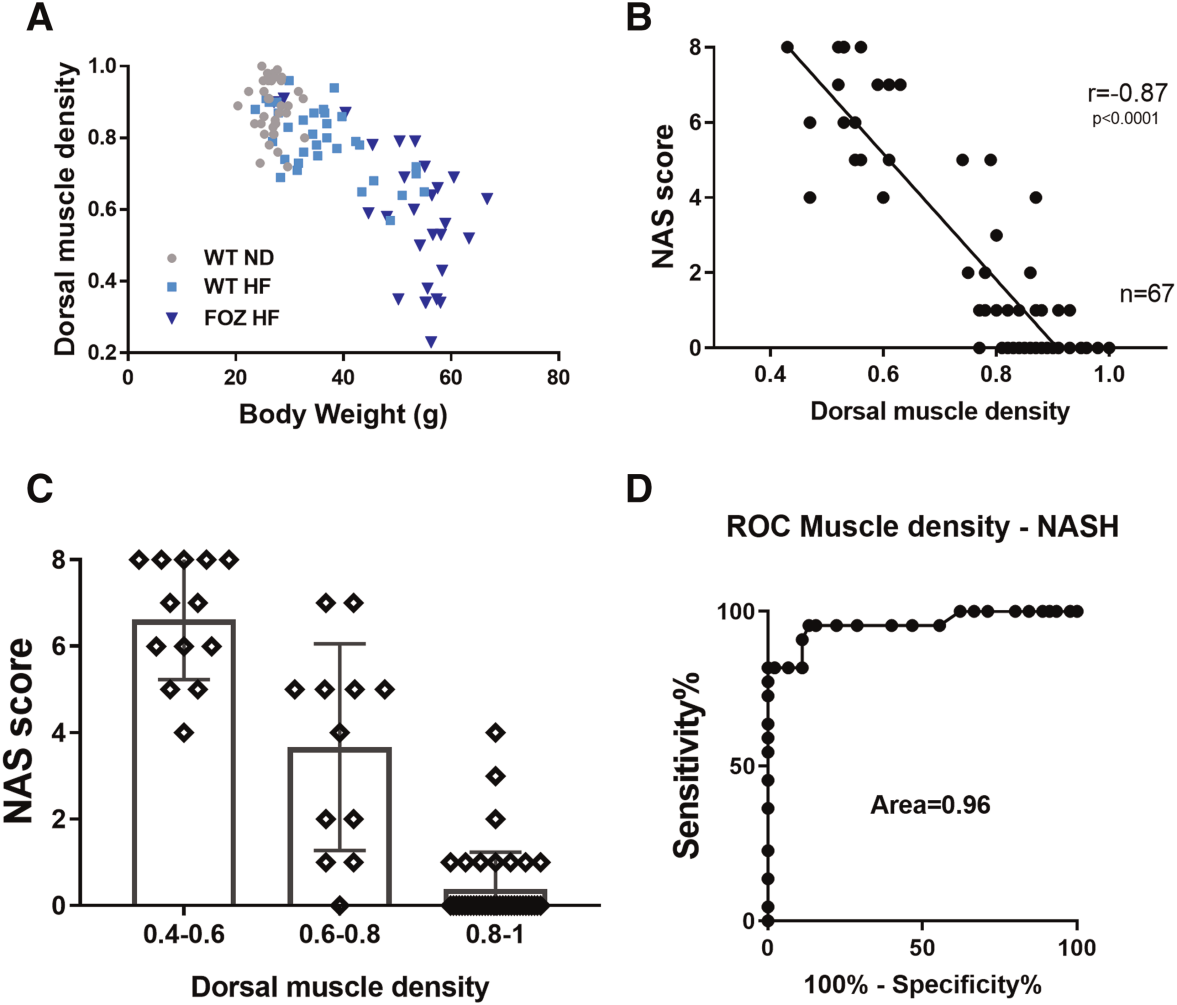
Myosteatosis strongly discriminates non‐alcoholic steatohepatitis (NASH) from non‐alcoholic fatty liver (NAFL) in preclinical model of NAFL disease (NAFLD). *(A)* Relationship between body weight and dorsal muscle density. Muscle density is linearly correlated with body weight in high‐fat (HF) diet‐fed wild‐type (WT) mice (WT HF) but not in HF diet‐fed fat aussie mice (FOZ HF), where there is a wide range of dorsal muscle density in a restricted range of body weight (*n* = 112). *(B)* Correlation between NAS score and dorsal muscle density (Pearson00027;s coefficient *r* = −0.87, *n* = 67, *P* 60; 0.0001). *(C)* Relationship between muscle density and NAS score. Muscle densities are subdivided into three sub‐groups: low (0.4–0.6), mild (0.6–0.8), and high (0.8–1) density (*n* = 67). *(D)* Receiver operating characteristic (ROC) curve to evaluate the performance of dorsal muscle density to diagnose NASH (NAS ≥ 3 with at least 1 point in each sub‐score) vs. no‐NASH; *n* = 67, area under the receiver operating characteristic (ROC) (AUROC) = 0.96, 95% confidence interval (CI) of 0.92–1, *P* 60; 0.0001. At a threshold of <0.805, dorsal muscle density had 95.45%% sensitivity (95% CI of 77.16–99.88%) and 86.67% specificity (95% CI of 73.21–94.95%) to identify NASH. All data are mean ± SD. W, weeks.

Myosteatosis was seen in three independent NASH rodent models (i.e. WT HF, WT HFF, and FOZ HF). In FOZ HF, myosteatosis could not be explained by the higher body weight as illustrated in *Figure* 6A, wherein animals exhibited a wide range of muscle densities (≃0.8 to 0.2) for a similar body weight range.

This, as well as the absence of relationship with visceral adiposity and insulin resistance, prompted us to propose that myosteatosis could be a marker to distinguish NASH from NAFL. We correlated muscle density and NAFLD severity, as histologically evaluated with the NAS score. In the longitudinal mice cohort (i.e. WT ND, WT HF, and FOZ HF at 0, 4, 8, 12, 20, and 34 weeks), dorsal muscle density strongly negatively correlated with the NAS score (*r* = −0.87) (*Figure* 6B). The correlation persisted when animals without NAFLD (i.e. NAS = 0) were excluded (*r* = −0.83). Strikingly, 95% of mice with a dorsal muscle density over 0.8 did not have NASH (39 out of 41), while 100% of mice with a muscle density 60;0.6 had NASH (*Figure* 6C). The likelihood ratio for having NASH with a muscle density 60;0.76 was 37. We then tested the diagnostic power of muscle density. Muscle density distinguishes NASH from NAFL and normal liver with a nearly maximum performance score [area under the receiver operating characteristic (ROC) (AUROC) = 0.96, *P* 60; 0.0001] (*Figure* 6D). This impressive diagnostic power was maintained when only animals with NAFLD (i.e. at least 1 point in steatosis sub‐score) were considered (AUROC = 0.93, *P* 60; 0.0001). An optimal muscle density cut‐off of 0.805 had a sensitivity and a specificity of 95.45% and 86.67%, respectively, to predict NASH.

## Discussion

An increasing body of clinical literature has linked skeletal muscle alterations with NAFLD presence and severity.^11^, ^14^, ^15^, ^16^, ^17^, ^18^, ^19^, ^20^, ^21^ However, most studies are cross‐sectional and do not allow for longitudinal assessment of skeletal muscle changes in relation with liver disease progression. Hence, whether sarcopenia and/or myosteatosis are mere consequences of NASH or whether their onset might precede or parallel NASH remain largely unknown. To answer these questions, we performed a longitudinal study in which we monitored muscle alterations in relation with liver disease progression in preclinical NAFLD models. We drew inspiration from the clinical gold standard^7^ and developed and validated a micro‐CT‐based methodology for specific measurement of muscle mass, myosteatosis, and liver steatosis *in vivo* in mice. The technique accurately measures muscle mass and muscle and liver fatty infiltration. Moreover, it has a very high throughput (2.5 min scanning time per animal), and post‐processing analysis is trivial. Previous studies have used micro‐CT to evaluate liver steatosis in mice,^35^, ^36^ but the muscle indices validated here represent seminal data with potential applications beyond the scope of liver studies. With this technique, we showed in three independent NAFLD/NASH rodent models that sarcopenia (as evidenced by a low muscle strength, but a not low muscle mass) is present in mice with fibrosing NASH, but not in those with early NASH or NAFL. Hence, among the NAFLD spectrum, only fibrosing NASH is associated with sarcopenia. These findings contrast with available clinical data.^14^, ^15^, ^16^, ^17^, ^18^, ^19^, ^20^, ^21^, ^37^, ^38^ It is however important to state that the vast majority of clinical sarcopenia studies scaled muscle mass on body weight or body mass index to compute sarcopenia indexes.^14^, ^15^, ^16^, ^17^, ^18^, ^19^, ^20^, ^21^, ^37^, ^38^ Because obesity *per se* is a well‐known risk factor for NAFLD,^3^ it is not surprising to find a low relative muscle mass when muscle mass is scaled on body weight or body mass index in NAFLD patients. Data reported by Peng *et al*.^39^ strongly support this proposal. When muscle mass was scaled on body weight, the odds ratio for having sarcopenia was 1.73 (95% confidence interval 1.31–2.28) in NAFLD patients. In contrast, when muscle mass was scaled on height, the odds ratio for having sarcopenia was 0.63 (95% confidence interval 0.46–0.87), supporting a lower risk for sarcopenia in NAFLD. Hence, clinical studies with muscle strength evaluation, gold standard techniques to measure muscle mass, and adequate data presentation are urgently needed to clarify the relationship between NAFLD and sarcopenia.^6^

Of note, FOZ HF had decreased muscle strength, but not a low muscle mass when compared with WT ND controls. Rather, we observed a non‐adaptation to increasing body weight (hence, low relative muscle mass). A plausible explanation could be that the chronic work load imposed by excess weight in obese mice (WT HF, WT HFF, and FOZ HF) may prevent absolute muscle mass loss and maintain muscle strength, at least up to a certain point.

In contrast to sarcopenia, myosteatosis was found in three independent preclinical NASH models, both in early and fibrosing NASH. Its degree was strongly correlated with liver disease activity, and the association was independent from visceral fat or insulin resistance. Because our data support that myosteatosis is model independent and is not entirely explained by weight gain or metabolic disorder, we hypothesized that it could represent a surrogate diagnostic marker for NASH. We stratified muscle density according to histological NAS (i.e. the gold standard to evaluate NASH activity). We found that the likelihood ratio for having NASH with a muscle density 60;0.76 was 37. We therefore tested the diagnostic power of muscle density (reflecting myosteatosis severity on a continuous scale) on the entire longitudinal cohort using ROC curve. Muscle density had an impressive diagnostic performance to discriminate NASH from NAFL or normal liver (AUROC 0.96, *n* = 67, *P* 60; 0.0001).

Muscle mass is evaluated in most clinical NAFLD studies because bioelectrical impedance analysis or dual X‐ray absorptiometry are readily available. However, these techniques do not allow for muscle fat evaluation. Conversely, studies that used CT scan or magnetic resonance imaging enable quantification of muscle fatty infiltration and suggested a plausible association between myosteatosis and NAFLD. For instance, Kitajima *et al*.^17^ reported an association between myosteatosis and NASH severity in NAFLD patients (*n* = 208). Recently, Tanaka *et al*.^21^ reported the presence of low muscle density in patients with NAFLD when compared with controls (total *n* = 632). Some very recent papers nicely complement these findings.^23^, ^40^ Nonetheless, because of the lack of liver biopsy^21^, ^23^, ^40^ or improper analysis,^17^ none explores the potential of myosteatosis to diagnose early NASH in a steatotic liver. Currently, early NASH diagnosis relies on a liver biopsy. However, this invasive procedure is not performed unless transaminases are perturbed or signs of advanced fibrosis are present, such as captured non‐invasively by Fibrosis‐4 (FIB‐4), NAFLD fibrosis score (NFS), or liver stiffness measurements.^41^ Notwithstanding, hepatic transaminases are not always elevated in patients with NASH,^42^ and the diagnostic power of non‐invasive scores or imaging for fibrosis detection is poor at early stages of the disease (60;F2).^41^ Hence, a substantial proportion of patients with NASH‐F0–1 are probably undiagnosed with current guidelines. These patients have an increased risk for liver disease progression.^3^ Moreover, they also are at risk of cardiovascular events and of hepatocellular carcinoma, even in the absence of fibrosis.^3^ Therefore, there is an urgent need for the development of new markers to detect patients with early NASH, as this may help identifying an at‐risk population on which to concentrate surveillance and treatment options.

Overall, our preclinical data pave the way for well‐designed prospective clinical study to determine whether the evaluation of myosteatosis could help stratify at‐risk patients for NASH among the two billion adults with NAFLD.^3^ Noteworthy, validated clinical tools to evaluate myosteatosis are readily available in research settings^43^ and in clinical routine, with point‐of‐care technique such as ultrasound,^44^ which are suitable for large population screening.

Whether myosteatosis is a mere signature of the metabolic syndrome or whether it drives a pathophysiological process for disease progression still needs to be determined.^19^, ^24^ Thus, myosteatosis and NASH may represent two manifestations of ectopic lipid storage and lipotoxicity. The skeletal muscle is now recognized as an endocrine organ *per se*, secreting myokines that act distantly.^45^ It is thus tempting to speculate that severe fat infiltration may modify the secretome of skeletal muscles and in turn influence liver disease progression. Although the exploration of mechanisms leading to myosteatosis and sarcopenia in NASH (such as specific muscle insulin resistance) and the plausible consequences on muscle secretome go beyond the diagnostic scope of this paper, investigating whether a muscle‐to‐liver axis operates in NAFLD pathophysiology^19^ could bring new mechanistic insights of high significance for therapeutic development.

To the best of our knowledge, this is the first study wherein the muscle compartment was investigated longitudinally, non‐invasively, and invasively with gold standard methods in three validated NAFLD models, with a complete validation of a novel non‐invasive methodology (micro‐CT). With this study, we believe to be the first to answer the long‐standing ‘egg or chicken’ sarcopenia paradigm in NAFLD.^25^ Indeed, our experimental data strongly support that there is no sarcopenia in mice with obesity, metabolic syndrome, and NAFL or early NASH. Rather, sarcopenia associates with active and fibrosing liver disease. In contrast, myosteatosis, an under‐explored muscle change with potential pathophysiological relevance for liver disease progression, is the earliest muscle alteration in NASH. It was consistently found in three independent preclinical models. Myosteatosis predicted NASH with an impressive diagnostic performance (AUROC = 0.96). Hence, our data call for exploiting muscle fat content for non‐invasive diagnosis of NASH in NAFLD. Despite validation in three mouse models, whether muscle undergoes modification according to a similar kinetics in human NAFLD/NASH is unknown. Longitudinal studies with appropriate methodology for muscle and liver assessment (i.e. imaging and liver biopsy) are now needed to determine whether the non‐invasive evaluation of myosteatosis has a diagnostic and/or a prognosis value in patients with NAFLD. Myosteatosis and NASH are associated with increased mortality in patients,^8^, ^46^ and each condition carries an increased risk for cardiovascular complications,^47^, ^48^, ^49^ reported as the first cause of mortality in NAFLD.^1^ Moreover, myosteatosis is associated with incident type 2 diabetes,^50^ a condition tightly linked with NAFLD, and predicts mortality in these patients.^51^ Thus, myosteatosis might not only represent an early marker of NASH but could also directly influence its outcome. Further studies are needed to clarify this issue and determine whether myosteatosis could become a new therapeutic target in NAFLD.

## Author contributions

M.N. and I.A.L. conceived and designed the study. M.N., G.V., and M.D.R. performed animal experiments. G.V. and M.N. designed micro‐CT protocols. O.S. and C.B. provided expert advice and support for muscle testing and IHC and morphometrical quantification. Y.H. and J.‐P.T. critically discussed study design, data, and manuscript for scientific content. M.N. and I.A.L. wrote the manuscript, with contributions from all authors. I.A.L. obtained funding.

## Conflict of interest

The authors declare that they have no conflict of interest in relation to this work to disclose.

## Funding

This work was supported by the PhD fellowship from FRIA (FNRS, Belgium) [grant number 31618719 (to M.N.)] and by the Fund for Scientific Medical Research (FNRS Belgium) [grant number T.0141.19 (to I.A.L.)].

## Supporting information


**Figure S1.** FOZ HF consumes more calories than WT ND and WT HF and had severe insulin‐resistance (a) Composition of standard chow (left) and high fat diet (right). (b) Food intake expressed in kcal/mice/day (*n* = 5–6 mice/group/time point, two‐way ANOVA). All data are mean±SEM.

**Figure S2.** FOZ ND exhibit the same liver and muscle phenotype than WT HF (a) Body weight, (b) Liver density, (c) Dorsal muscle area (L4 and L5 averaged), (d) Dorsal muscle density (L4 and L5 averaged) measured *in‐vivo* by micro‐CT and (e) Grip strength of WT ND, WT HF, FOZ ND and FOZ HF at different time point until W34 (*n* = 5–19/group/time point, two‐way ANOVA, $ = FOZ HF significantly different from WT ND # = WT HF significantly different from WT ND, 38; = FOZ HF significantly different than WT HF, * = FOZ HF significantly different from WT HF). (two‐way ANOVA). All data are mean±SEM.

**Figure S3.** FOZ HF mice have visible fibrosis from W20 on (a) Left, representative histological picture of sirius red staining in 34 W FOZ HF (scale bar = 125 μm). Right, representative image of mask used for automated analysis of fibrosis area. (b) Fibrosis area on entire liver sections (*n* = 3–4/group). Line,median value; box, 25%–75% percentile; whiskers, min and max, one‐way ANOVA. **p 60; 0.01, ****p 60; 0.0001

**Figure S4.** *in‐vivo* 3D whole body acquisition with micro‐CT. Whole body composition of WT ND, WT HF and FOZ HF at 0 W, 12 W and 34 W. Density‐based colour‐scale (pinkish to red = high density values identifying lean tissues, turquoise to green = low density values identifying fat).

**Figure S5.** *in‐vivo* 3D reconstruction of liver and dorsal muscle with micro‐CT. Liver and dorsal muscle reconstruction of WT ND, WT HF and FOZ HF at 0 W, 12 W and 34 W. Density‐based colour‐scale (red = high density value representing lean mass, yellow = low density value representing fat infiltration). *Note: This figure illustrates fatty infiltration in liver and muscles, thus all items have similar colour‐scale, but not size‐scale*.

**Figure S6.** FOZ HF fail to adapt their muscle mass according to body weight gain. (a) Dorsal muscle area relative to body weight (*n* = 5–19/group/time point, two‐way ANOVA). (b) Gastrocnemius muscle weight at 0 W, 12 W and 34 W in WT ND, WT HF and FOZ HF (n = 5–9/group/time point, one‐way ANOVA). All data are mean±SEM.

**Figure S7.** WT HFF exhibits key NASH features, but remain metabolically comparable with WT HF. (a) Food intake expressed in kcal/mice/day (*n* = 4–11 mice/group/time point, student t test). (b) Fasting glycaemia over the study period (n = 4–11 mice/group/time point, two‐way ANOVA). (c) and (d) Representative liver histology in WT HF and WT HFF with H38;E staining (c) and F4:80 IHC (d) with respective automated computerized quantification (Biocellvia, Marseille, France).

**Table S1.** Muscle density is independently associated with NASH.
