# Supplementary figures and images for: Myosteatosis rather than sarcopenia associates with non‐alcoholic steatohepatitis in non‐alcoholic fatty liver disease preclinical models
Source: J Cachexia Sarcopenia Muscle. 2020 Nov 26;12(1):144–58. doi: 10.1002/jcsm.12646 (PMC7890270; doi:10.1002/jcsm.12646)

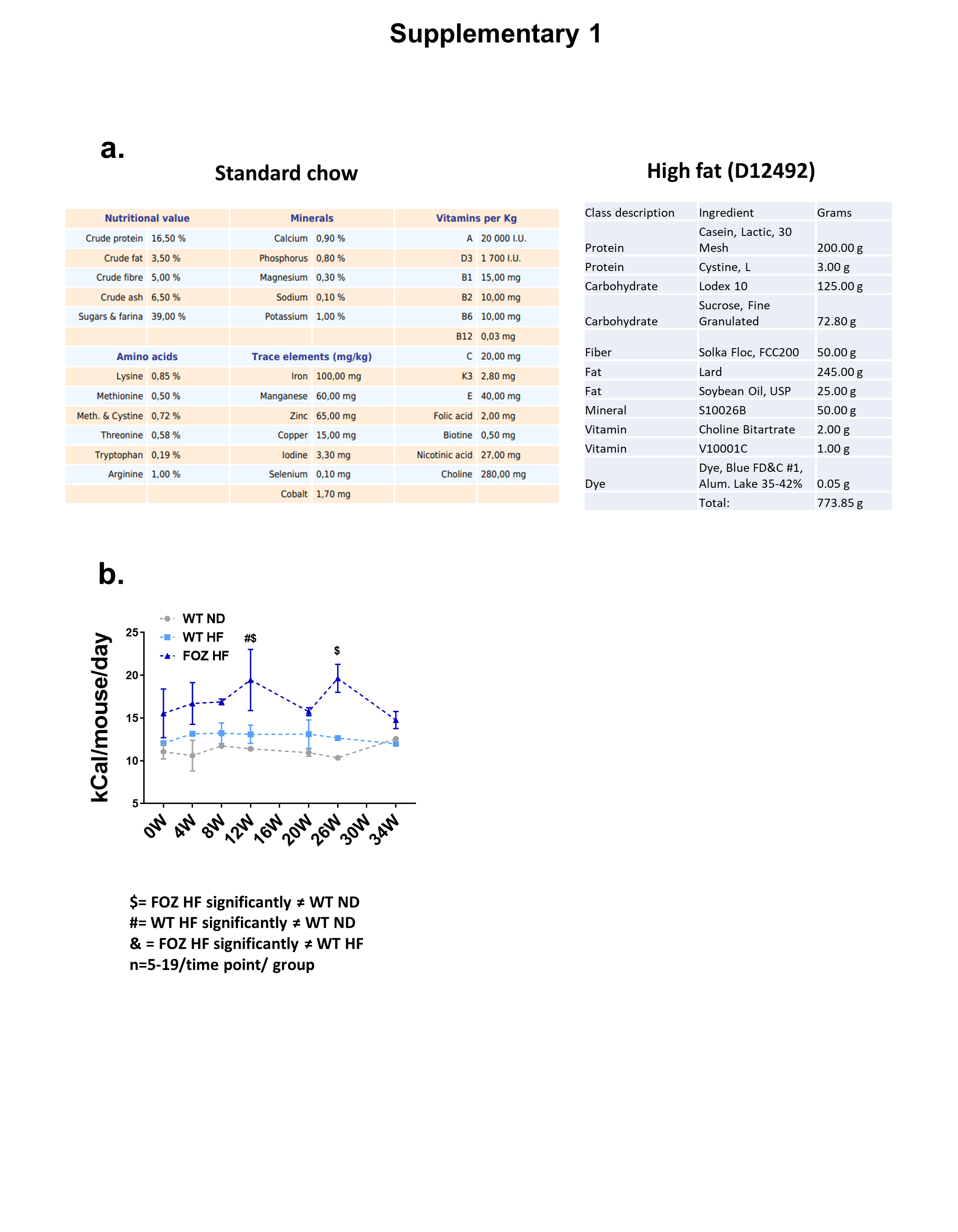

Supplement: Supplementary file 1 — Figure S1. FOZ HF consumes more calories than WT ND and WT HF and had severe insulin‐resistance (a) Composition of standard chow (left) and high fat diet (right). (b) Food intake expressed in kcal/mice/day (n = 5–6 mice/group/time point, two‐way ANOVA). All data are mean±SEM. [file JCSM-12-144-s001.TIF]

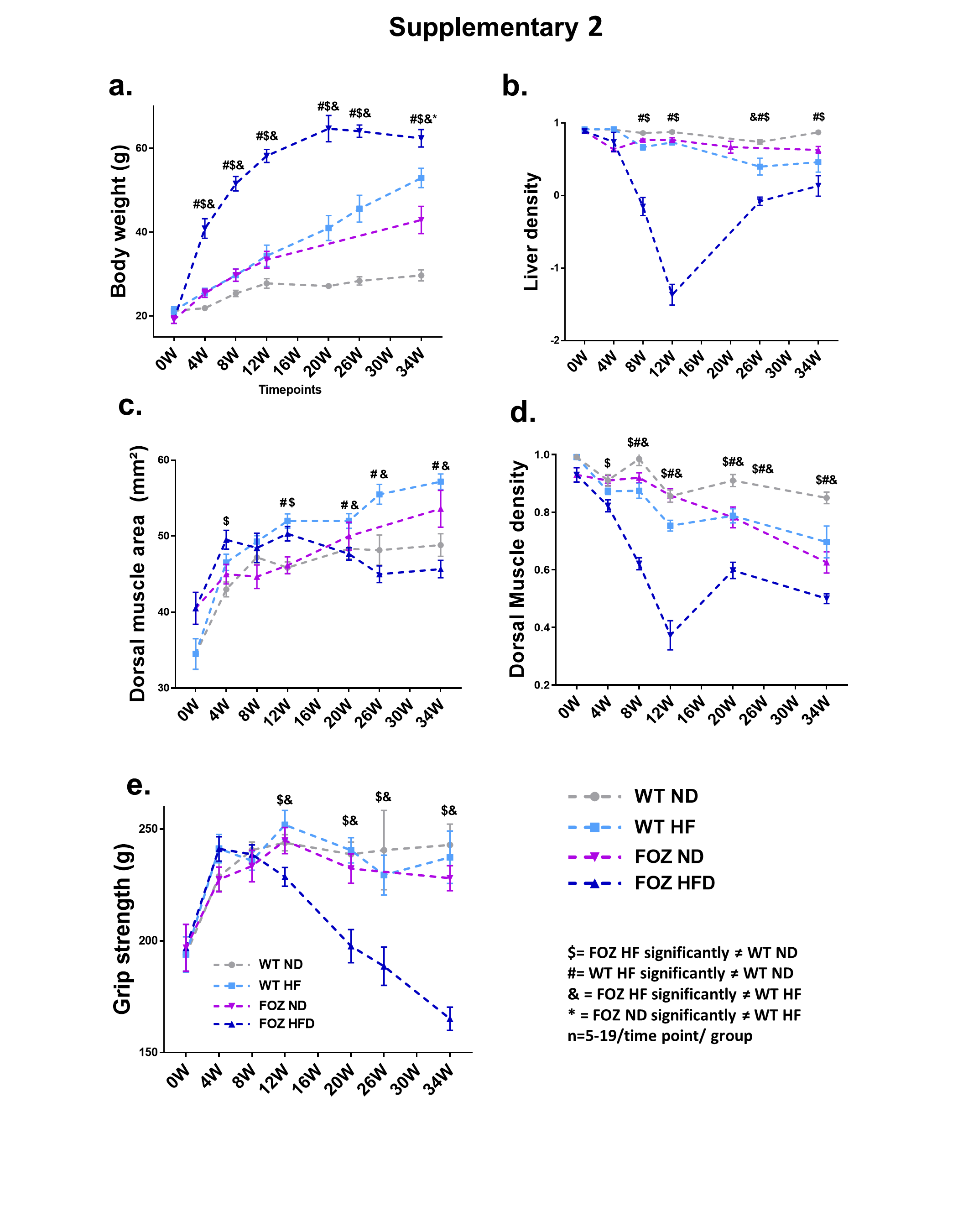

Supplement: Supplementary file 2 — Figure S2. FOZ ND exhibit the same liver and muscle phenotype than WT HF (a) Body weight, (b) Liver density, (c) Dorsal muscle area (L4 and L5 averaged), (d) Dorsal muscle density (L4 and L5 averaged) measured in‐vivo by micro‐CT and (e) Grip strength of WT ND, WT HF, FOZ ND and FOZ HF at different time point until W34 (n = 5–19/group/time point, two‐way ANOVA, $ = FOZ HF significantly different from WT ND # = WT HF significantly different from WT ND, 38; = FOZ HF significantly different than WT HF, * = FOZ HF significantly different from WT HF). (two‐way ANOVA). All data are mean±SEM. [file JCSM-12-144-s002.TIF]

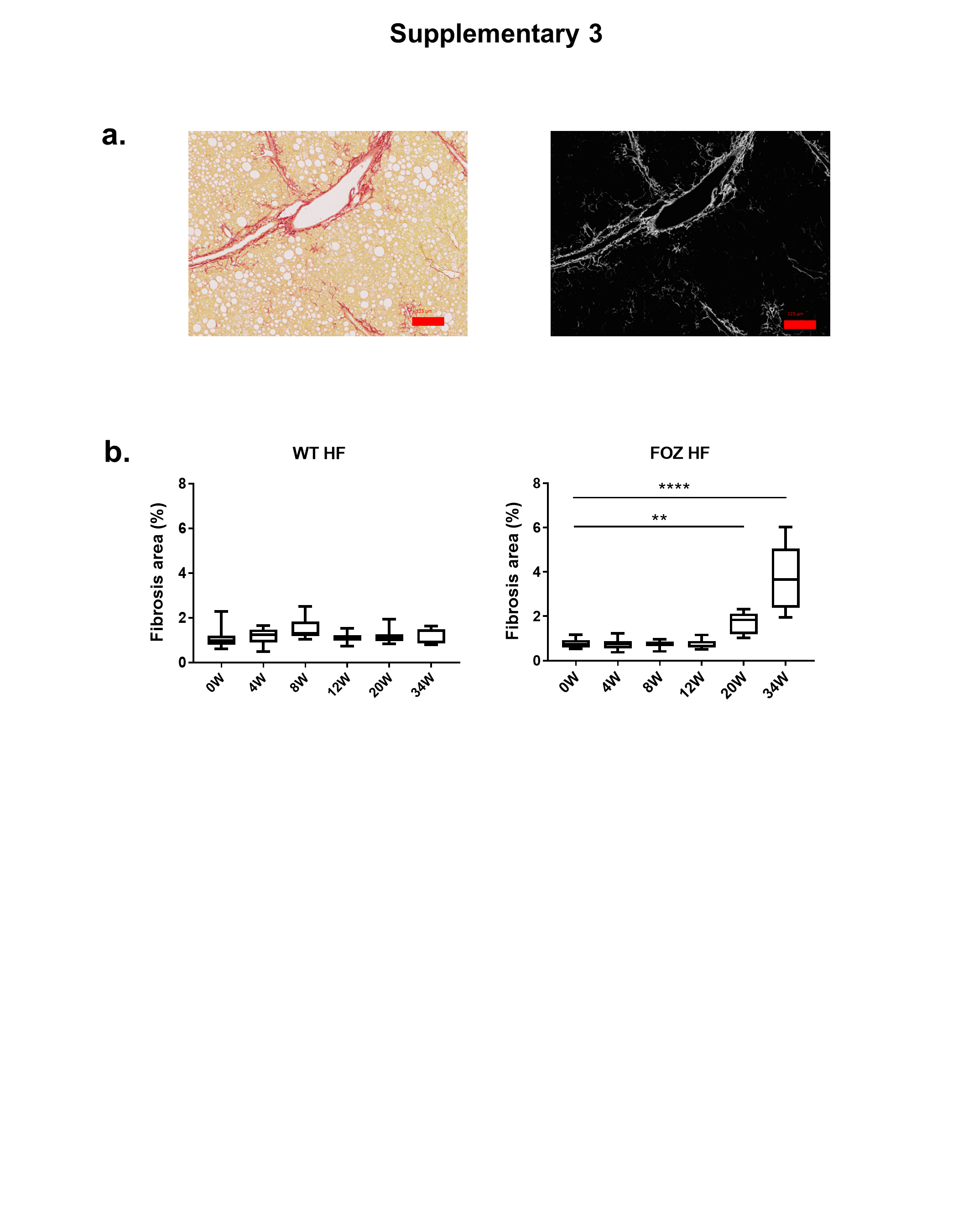

Supplement: Supplementary file 3 — Figure S3. FOZ HF mice have visible fibrosis from W20 on (a) Left, representative histological picture of sirius red staining in 34 W FOZ HF (scale bar = 125 μm). Right, representative image of mask used for automated analysis of fibrosis area. (b) Fibrosis area on entire liver sections (n = 3–4/group). Line,median value; box, 25%–75% percentile; whiskers, min and max, one‐way ANOVA. **p 60; 0.01, ****p 60; 0.0001 [file JCSM-12-144-s003.TIF]

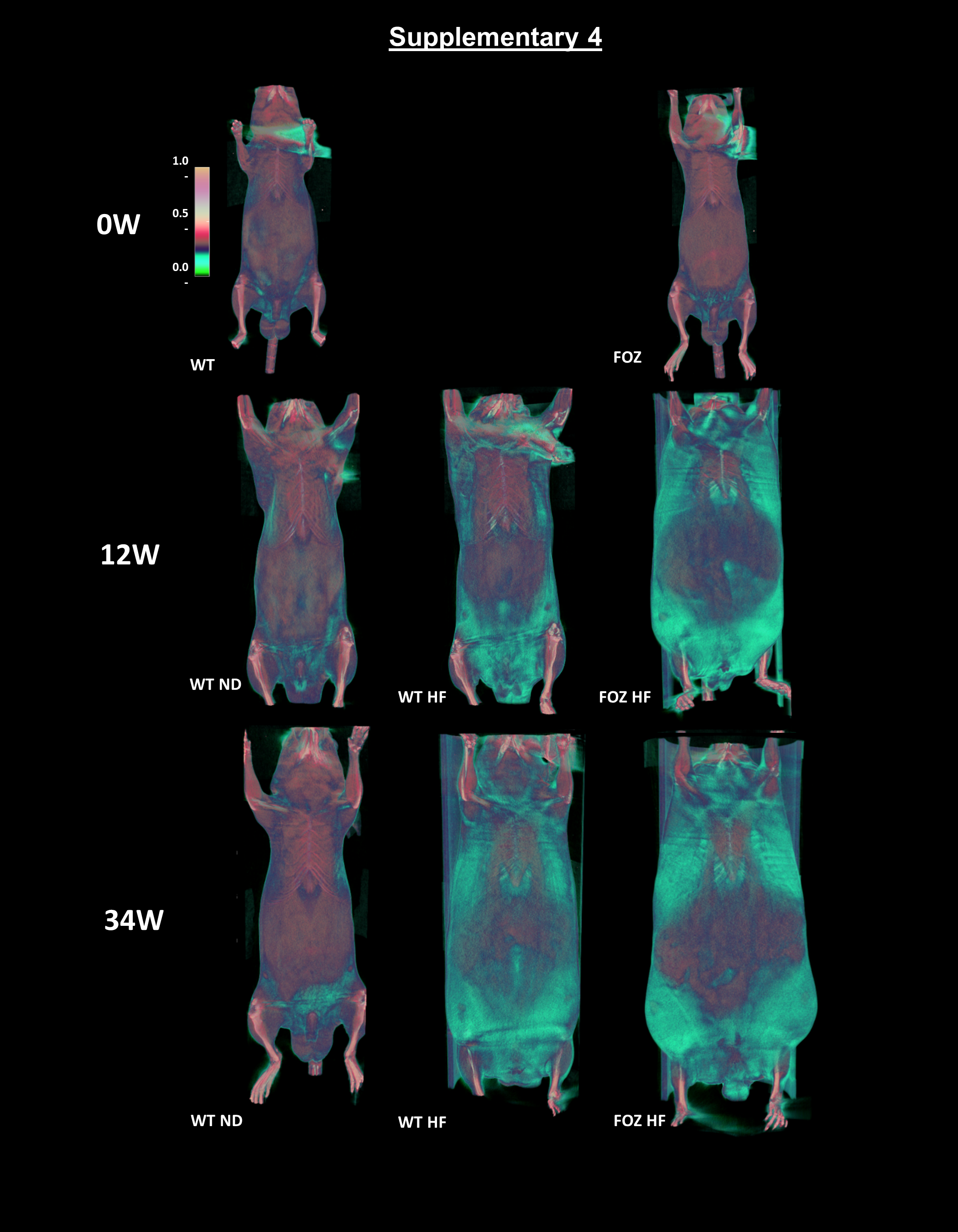

Supplement: Supplementary file 4 — Figure S4. in‐vivo 3D whole body acquisition with micro‐CT. Whole body composition of WT ND, WT HF and FOZ HF at 0 W, 12 W and 34 W. Density‐based colour‐scale (pinkish to red = high density values identifying lean tissues, turquoise to green = low density values identifying fat). [file JCSM-12-144-s004.TIF]

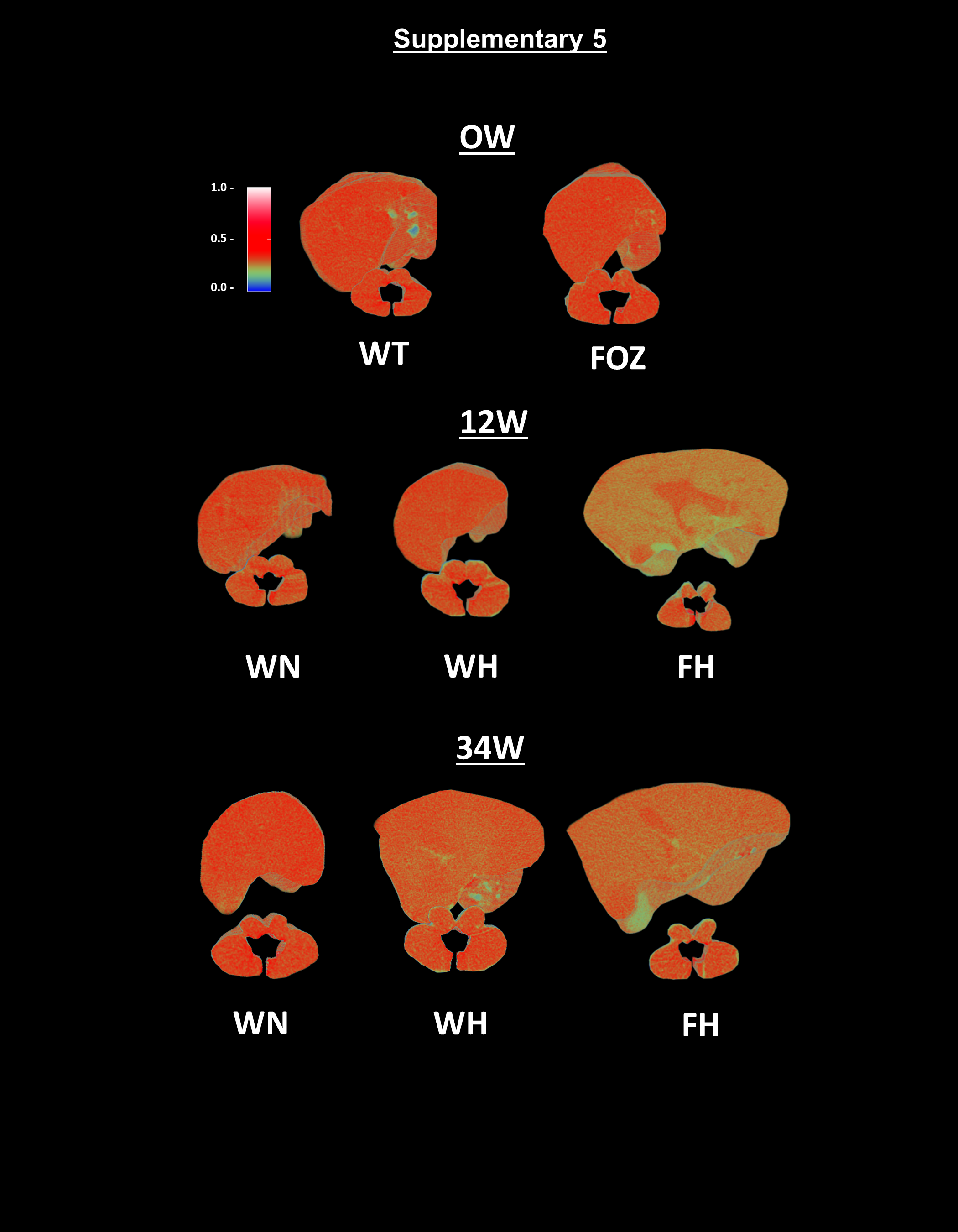

Supplement: Supplementary file 5 — Figure S5. in‐vivo 3D reconstruction of liver and dorsal muscle with micro‐CT. Liver and dorsal muscle reconstruction of WT ND, WT HF and FOZ HF at 0 W, 12 W and 34 W. Density‐based colour‐scale (red = high density value representing lean mass, yellow = low density value representing fat infiltration). Note: This figure illustrates fatty infiltration in liver and muscles, thus all items have similar colour‐scale, but not size‐scale. [file JCSM-12-144-s005.TIF]

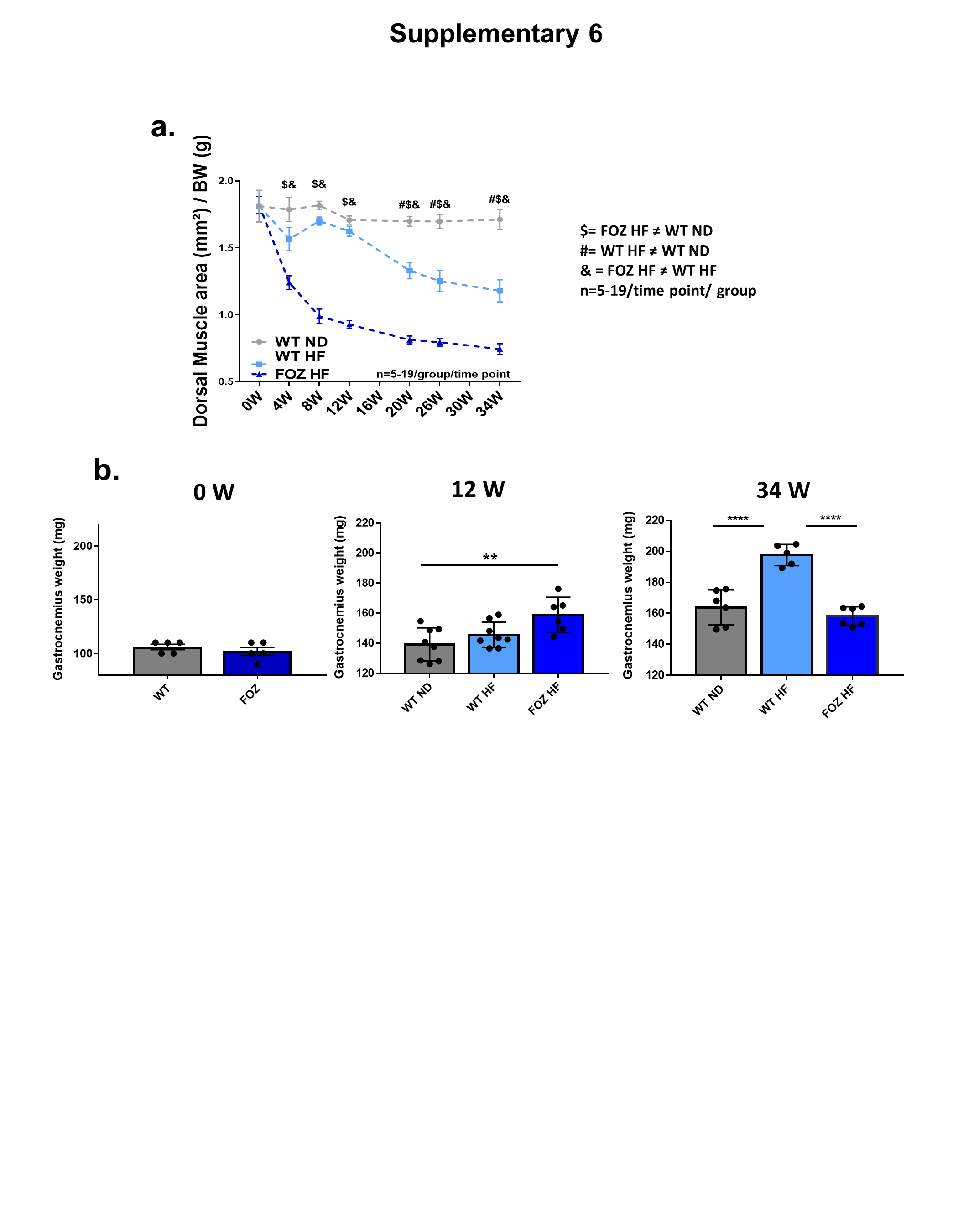

Supplement: Supplementary file 6 — Figure S6. FOZ HF fail to adapt their muscle mass according to body weight gain. (a) Dorsal muscle area relative to body weight (n = 5–19/group/time point, two‐way ANOVA). (b) Gastrocnemius muscle weight at 0 W, 12 W and 34 W in WT ND, WT HF and FOZ HF (n = 5–9/group/time point, one‐way ANOVA). All data are mean±SEM. [file JCSM-12-144-s006.TIF]

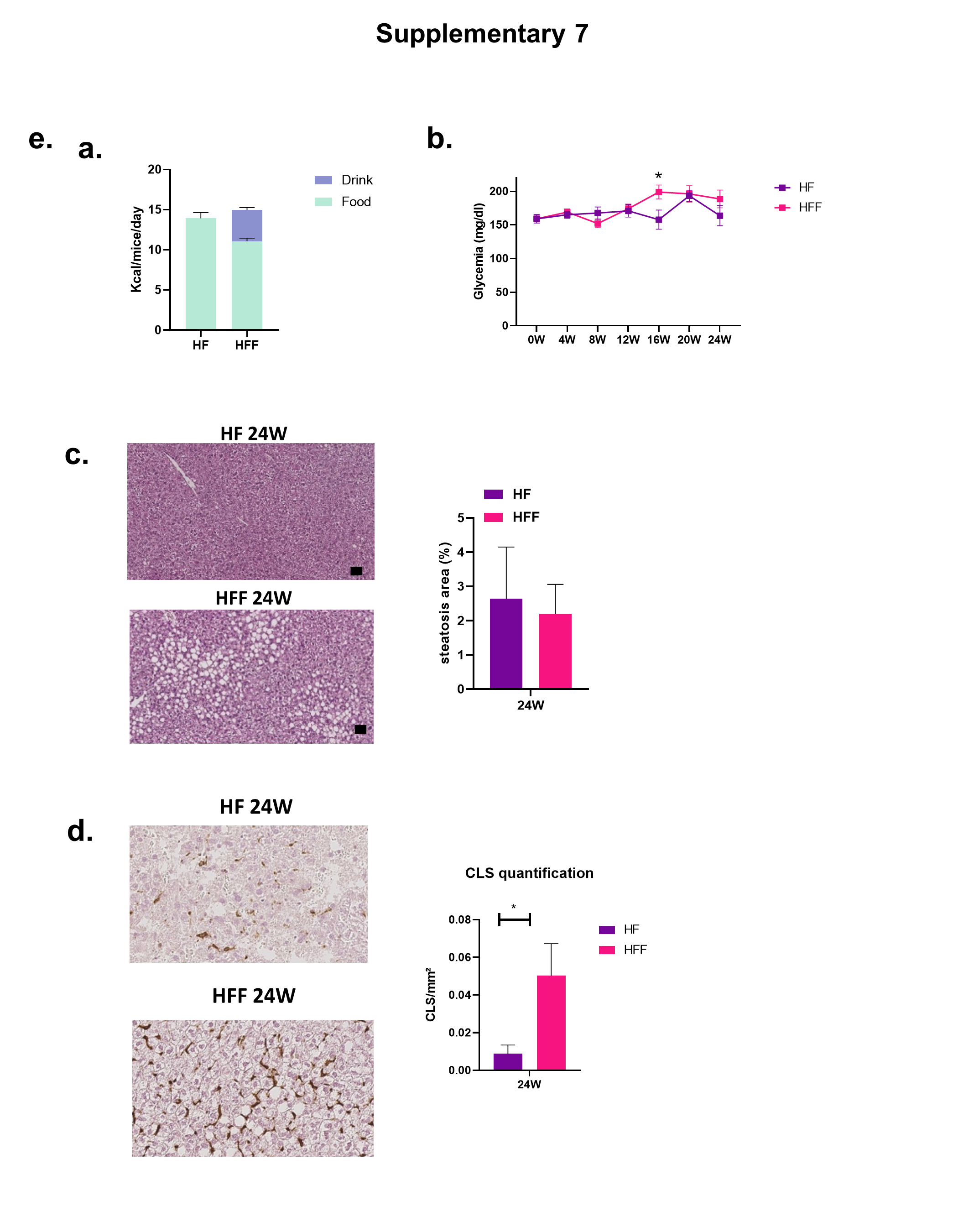

Supplement: Supplementary file 7 — Figure S7. WT HFF exhibits key NASH features, but remain metabolically comparable with WT HF. (a) Food intake expressed in kcal/mice/day (n = 4–11 mice/group/time point, student t test). (b) Fasting glycaemia over the study period (n = 4–11 mice/group/time point, two‐way ANOVA). (c) and (d) Representative liver histology in WT HF and WT HFF with H38;E staining (c) and F4:80 IHC (d) with respective automated computerized quantification (Biocellvia, Marseille, France). [file JCSM-12-144-s007.TIF]

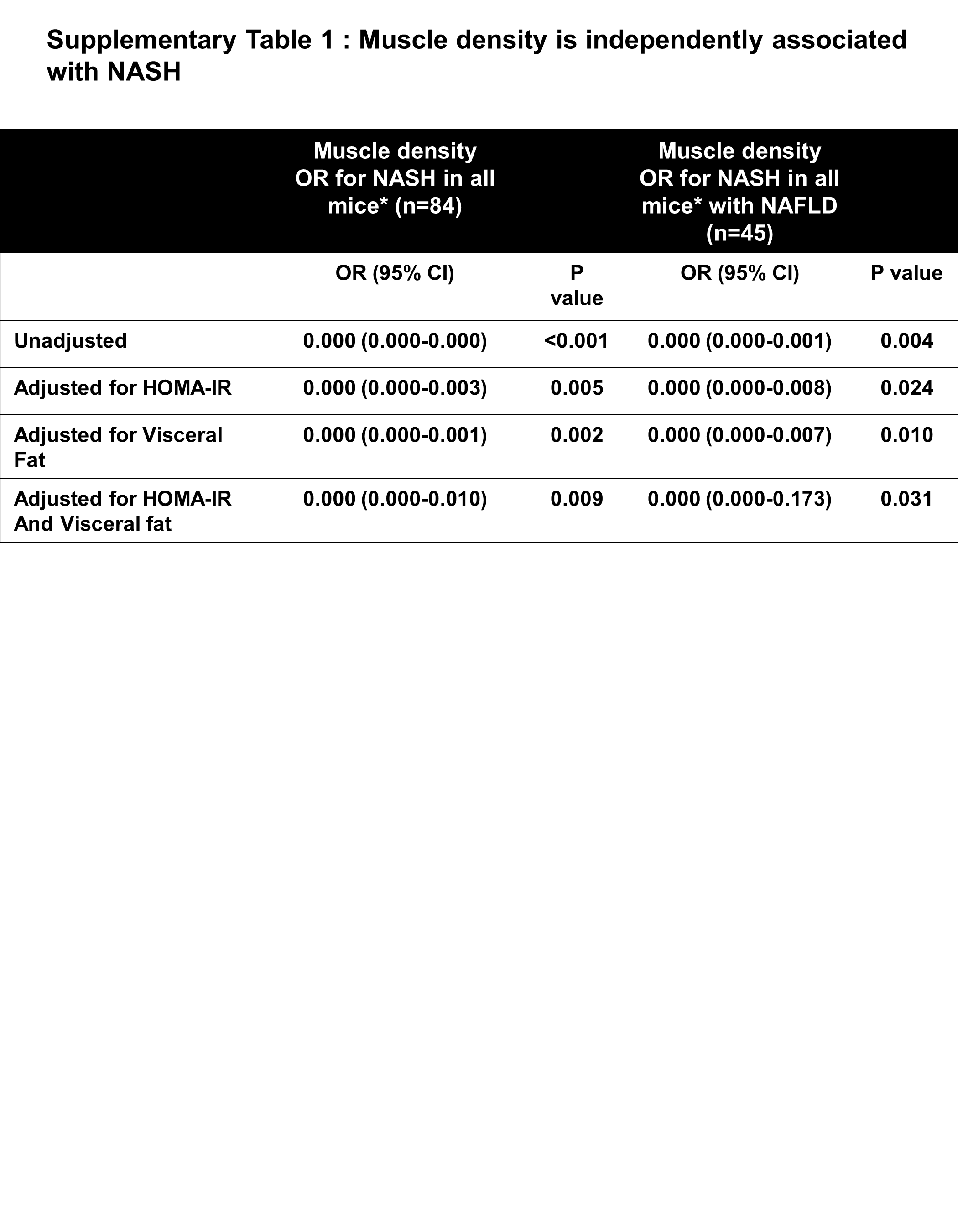

Supplement: Supplementary file 8 — Table S1. Muscle density is independently associated with NASH. [file JCSM-12-144-s008.TIF]
